# Endothelial α6β4 integrin protects during experimental autoimmune encephalomyelitis-induced neuroinflammation by maintaining vascular integrity and tight junction protein expression

**DOI:** 10.1186/s12974-017-0987-2

**Published:** 2017-11-09

**Authors:** Jennifer V. Welser, Sebok K. Halder, Ravi Kant, Amin Boroujerdi, Richard Milner

**Affiliations:** Department of Molecular Medicine, The Scripps Research Institute, 10550 North Torrey Pines Road, MEM-132, La Jolla, CA 92037 USA

**Keywords:** Endothelial, Extracellular matrix, Laminin, Integrin, Blood-brain barrier, Vascular

## Abstract

**Background:**

Extracellular matrix (ECM) proteins play critical functions regulating vascular formation and function. Laminin is a major component of the vascular basal lamina, and transgenic mice deficient in astrocyte or pericyte laminin show defective blood-brain barrier (BBB) integrity, indicating an important instructive role for laminin in cerebral blood vessels. As previous work shows that in the normal brain, vascular expression of the laminin receptor α6β4 integrin is predominantly restricted to arterioles, but induced on all vessels during neuroinflammation, it is important to define the role of this integrin in the maintenance of BBB integrity.

**Methods:**

α6β4 integrin expression was analyzed using dual immunofluorescence (dual-IF) of brain sections taken from the mouse model of multiple sclerosis, experimental autoimmune encephalomyelitis (EAE). To investigate the role of endothelial α6β4 integrin, transgenic mice lacking β4 integrin in endothelial cells (β4-EC-KO) and wild-type (WT) littermates were subject to EAE, and clinical score and various neuropathological parameters were examined by immunofluorescence. In addition, β4 integrin null brain endothelial cells (BECs) were examined in culture for expression of tight junction proteins using immunocytochemistry and flow cytometry.

**Results:**

Cerebrovascular expression of β4 integrin was markedly upregulated during EAE progression, such that by the acute stage of EAE (day 21), the vast majority of blood vessels expressed β4 integrin. In the EAE model, while the β4-EC-KO mice showed the same time of disease onset as the WT littermates, they developed significantly worse clinical disease over time, resulting in increased clinical score at the peak of disease and maintained elevated thereafter. Consistent with this, the β4-EC-KO mice showed enhanced levels of leukocyte infiltration and BBB breakdown and also displayed increased loss of the endothelial tight junction proteins claudin-5 and ZO-1. Under pro-inflammatory conditions, primary cultures of β4KO BECs also showed increased loss of claudin-5 and ZO-1 expression.

**Conclusions:**

Taken together, our data suggest that α6β4 integrin upregulation is an inducible protective mechanism that stabilizes the BBB during neuroinflammatory conditions.

## Background

Multiple sclerosis (MS) is a chronic inflammatory disease resulting in demyelination and degeneration of axons in the central nervous system (CNS) [1, 2]. While the precise trigger of MS remains elusive, it is characterized pathologically by multiple inflammatory lesions of white matter that are separated in time and space. Though most of the damage is caused by infiltrating leukocytes, strong evidence suggests that alterations in vascular properties at an early stage of disease onset play a central role in the initiation and/or maintenance of this pathology by permitting inflammatory leukocytes to enter the CNS [3–6]. Blood vessels in the CNS are unique in forming the blood-brain barrier (BBB), which confers high electrical resistance and low permeability properties, thus protecting neural cells from potentially harmful blood components [7–11]. The molecular basis of the BBB depends on (i) inter-endothelial tight junction proteins, (ii) endothelial adhesion to the extracellular matrix (ECM) proteins of the underlying vascular basal lamina, and (iii) the influence of astrocyte end-feet and pericytes [11–14].

ECM proteins play critical functions shaping vascular formation during development and in the adult [15–19], making it a high priority to define the role of specific ECM proteins and their integrin receptors in regulating vascular remodeling and BBB integrity. The laminin receptor, α6β4 integrin, warrants special attention for several reasons. First, laminin is a major component of the basal lamina of blood vessels, and transgenic mice deficient in astrocyte or pericyte laminin show defective BBB integrity [20, 21]. Second, in contrast to other laminin receptors such as α6β1 integrin and dystroglycan, in the normal brain, α6β4 integrin shows a limited distribution pattern, with expression restricted to arterioles [22]. Third, previous studies show that the number of cerebral vessels expressing α6β4 integrin is strongly increased in two models of neuroinflammation (GFAP-IL6 transgenic mice and a cerebral aneurysm model) [23, 24]. Fourth, in skin, α6β4 integrin plays a critical role in promoting mechanical stability and barrier integrity [25–27]. Taken together, these data raise the possibility that α6β4 integrin may play a protective role in the regulation of BBB integrity.

Previous studies have suggested a role for the β1 class of integrins in modulation of BBB integrity [14], but so far, no one has defined a function for any one specific integrin. In light of the dynamic regulation of α6β4 integrin expression on cerebral blood vessels during neuroinflammation [23, 24], and its essential role in the maintenance of epidermal integrity in skin [25–27], the goal of this study was to examine the contribution of α6β4 integrin to BBB integrity by answering three main questions. First, how is endothelial β4 integrin expression regulated in the mouse model of MS, experimental autoimmune encephalomyelitis (EAE)? Second, how does genetic deletion of endothelial β4 integrin (using transgenic β4-EC-KO mice) impact progression of EAE and its underlying pathology? Third, how does absence of endothelial β4 integrin affect BBB integrity and endothelial expression of tight junction proteins?

## Methods

### Animals

The studies described have been reviewed and approved by The Scripps Research Institute Institutional Animal Care and Use Committee. The β4 integrin^flox/flox^ transgenic mice were a kind gift from Dr. Laura Feltri (University of Buffalo). The generation of Tie2-Cre, nestin-Cre, and β4 integrin^flox/flox^ (β4 integrin^f/f^) strains of mice and genotyping protocols have all been described previously [28–30]. All strains were backcrossed > 10 times onto the C57BL/6 background and maintained under specific pathogen-free conditions in the closed breeding colony of The Scripps Research Institute (TSRI).

### Experimental autoimmune encephalomyelitis (EAE)

EAE was performed using a protocol and materials provided by Hooke Laboratories (Lawrence, MA). Briefly, 8–10-week-old β4-EC-KO or WT littermate control (β4^flox/wt^) female mice were immunized subcutaneously with 100 μl of 1 mg/ml MOG_33–35_ peptide emulsified in complete Freud’s adjuvant (CFA) containing 2 mg/ml *Mycobacterium tuberculosis* in both the base of the tail and upper back. In addition, on days 0 and 1, the mice also received an intraperitoneal injection of 200 ng pertussis toxin. The control mice received CFA containing no MOG peptide. This protocol leads to robust induction of clinical EAE on days 12–14 following immunization [31, 32]. The animals were monitored daily for clinical signs and scored as follows: 0—no symptoms, 1—flaccid tail, 2—paresis of hind limbs, 3—paralysis of hind limbs, 4—quadriplegia, and 5—death. The control and EAE mice were euthanized at different time points of disease, including 0, 4, 7, 14, 21, and 35 days. Most histological studies were performed on tissue obtained at the 21-day time point, corresponding to the acute symptomatic stage of disease.

### Immunohistochemistry and antibodies

Immunohistochemistry was performed on 10-μm frozen sections of cold phosphate buffer saline (PBS)-perfused tissues as described previously [33]. Antibodies reactive for the following antigens were used in this study: rat monoclonals reactive to CD31 (MEC13.3), β4 integrin (346-11A), MHC class II (M5/114.15.2), CD45, Mac-1 (M1/70), all from BD Pharmingen (La Jolla, CA); mouse monoclonal α-SMA-Cy3 conjugate (1A4) from Sigma (St. Louis, MO); rabbit polyclonals reactive to claudin-5 and ZO-1 from Invitrogen (Carlsbad, CA); and fibrinogen from Millipore (Temecula, CA). Secondary antibodies used included Cy3-conjugated anti-rat and anti-rabbit from Jackson Immunoresearch (West Grove, PA) and anti-rat Alexa Fluor 488 and anti-rabbit Alexa Fluor 568 from Invitrogen (Carlsbad, CA).

### Image analysis

Images were taken using a ×20 objective on a Zeiss Imager M1.m. microscope. Analysis was performed specifically in the medulla oblongata region of the brain. For each antigen, four images were taken per region at ×20 magnification, and a minimum of three sections per brain were analyzed to calculate the mean for each subject. All data analysis was performed using NIH ImageJ software. This analysis was performed using four animals of each genotype per condition per experiment, and the results were expressed as the mean ± SEM. Statistical significance was assessed by using the Student’s *t* test, in which *p* < 0.05 was defined as statistically significant. Blood-brain barrier (BBB) integrity was evaluated by measuring extravascular leakage of fibrinogen, in which the total area of fibrinogen staining per field of view (FOV) was divided by the total vascular area (CD31 signal) in the same FOV. Vascular expression of tight junction proteins was evaluated by measuring the total claudin-5 or ZO-1 area per FOV divided by the total vascular area for the same FOV.

### Cell culture

Pure cultures of primary mouse brain endothelial cells (BECs) derived from the β4-EC-KO or littermate control mice were prepared as previously described [34, 35]. Briefly, the brains were removed from the 8-week-old mice, minced, dissociated for 1 h in papain and DNase I, and centrifuged through 22% BSA to remove myelin, and endothelial cells were cultured in endothelial cell growth media (ECGM) consisting of Hams F12, supplemented with 10% FBS, heparin, ascorbic acid, L-glutamine, penicillin/streptomycin (all from Sigma), and endothelial cell growth supplement (ECGS) (Upstate Cell Signaling Solutions, Lake Placid, NY), on type I collagen (Sigma)-coated six-well plates. To obtain BECs, puromycin (4 μg/ml, Alexis GmbH, Grunberg, Germany) was included in culture media between days 1–3 to remove contaminating cell types. Endothelial cell purity was > 99% as determined by CD31 in flow cytometry. For all experiments, BECs were used only for the first passage.

### Immunofluorescence of primary BECs

Coverslips were coated with laminin (Sigma, 10 μg/ml) for 2 h at 37 °C, and BECs from the β4-EC-KO or wild-type littermate mice were plated onto them. Upon reaching confluence, the cells were treated with either 10 ng/ml TNF-α or IFN-γ (both from R&D, Minneapolis, MN) for 48 h. After this time, the cells were fixed for 5 min in acetone/methanol (1:1) at − 20 °C and blocked for 30 min in 5% normal goat serum in PBS containing 0.2% triton X-100 (Sigma) to permeabilize the cells (maintained in all incubation steps thereafter). The cells were then incubated with a rabbit polyclonal anti-claudin-5 antibody (Invitrogen) for 1 h followed by anti-rabbit-Cy3 secondary (Jackson Immunoresearch) for 1 h, labeled with the nuclear marker Hoechst (Sigma) for 5 min before being washed and mounted on glass slides.

### Flow cytometry

Brain endothelial cell (BEC) expression of claudin-5 and ZO-1 was examined as described previously [36]. Briefly, BECs derived from the β4-EC-KO or littermate control mice were first passaged onto laminin-coated six-well plates. Upon reaching confluence, the cells were treated with either 10 ng/ml TNF-α or IFN-γ (both from R&D) for 48 h and then removed and cellular expression of claudin-5 and ZO-1 analyzed by flow cytometry using rabbit polyclonal anti-claudin-5 or anti-ZO-1 antibodies followed by anti-rabbit Alex Fluor 488 secondary (both from Invitrogen). As the claudin-5 and ZO-1 antibodies are directed against intracellular epitopes, the cells were first fixed and then permeabilized using the Cytofix/Cytoperm kit (BD Pharmingen) and all subsequent incubations were performed in the cytoperm buffer. The fluorescent intensity of labeled cells was analyzed with a Becton Dickinson FACScan machine, with 10,000 events captured for each condition. Each experiment was repeated a minimum of four times and the data expressed as mean ± SEM fluorescent intensity. Statistical significance was assessed by using the Student’s paired *t* test, in which *p* < 0.05 was defined as statistically significant.

## Results

### β4 integrin is strongly upregulated on brain endothelial cells during neuroinflammation in the EAE model

**Fig. 1:**
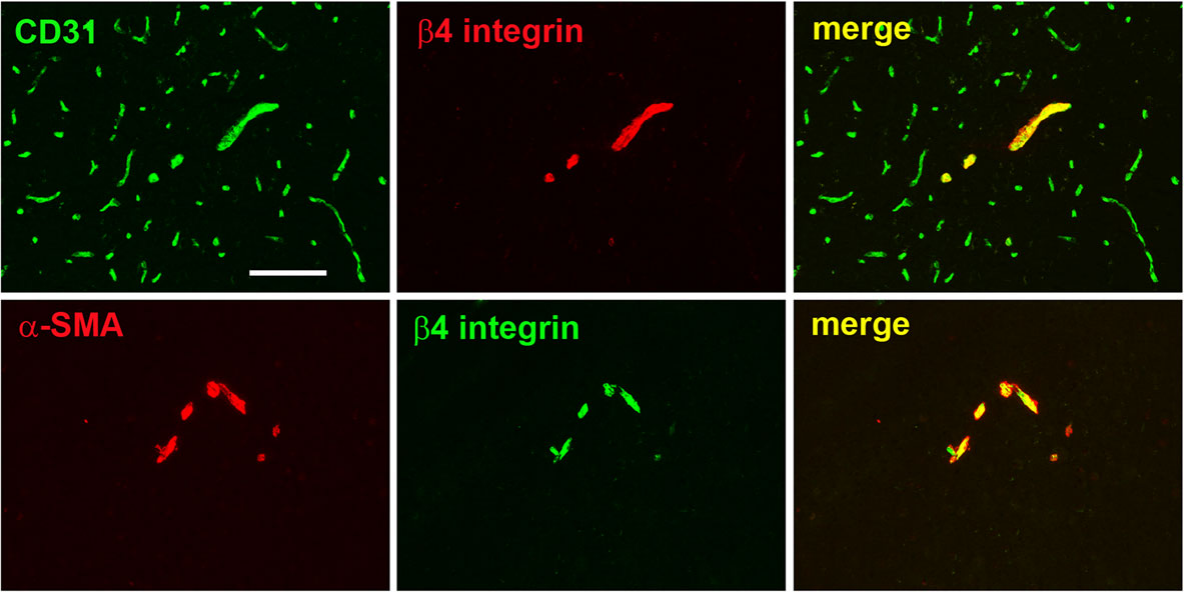
Characterization of β4 integrin expression on blood vessels in the normal brain. Top panel shows dual-IF on frozen sections of the medulla oblongata from adult mice using antibodies specific for the endothelial marker CD31 (AlexaFluor-488, green) and β4 integrin (Cy3, red). Lower panel shows dual-IF using antibodies specific for smooth muscle cell marker α-SMA (Cy3, red) and β4 integrin (AlexaFluor-488, green). Scale bar = 100 μm. Note that β4 integrin was expressed by only a fraction of CD31-positive vessels, but co-localized strongly with α-SMA

Extracellular matrix (ECM) proteins play critical functions influencing vascular cell behavior both during development and in the adult [15–19]. In light of the key role of laminin in contributing to blood-brain barrier (BBB) integrity of cerebral blood vessels [20, 21], it is a high priority to define the role of specific integrin laminin receptors in mediating these effects. The best-defined laminin receptors are the α6 integrins, α6β1 and α6β4, functional heterodimers comprising the α6 integrin subunit coupled with either the β1 or β4 integrin subunits, respectively [37]. Interestingly, in a previous study, we found that while the α6 and β1 integrin subunits are expressed universally by all cerebral blood vessels, expression of the β4 integrin subunit is restricted predominantly to endothelial cells within arterioles [22]. As shown in Fig. 1 (top row), dual immunofluorescence (dual-IF) with the endothelial marker CD31 shows that β4 integrin is expressed on only a small fraction of cerebral blood vessels, which tend to be larger diameter vessels. The lower row of Fig. 1 reveals that β4 integrin co-localizes strongly with α-smooth muscle actin (α-SMA), a marker of arterial vessels, indicating that β4 integrin is expressed predominantly in cerebral arterioles.

**Fig. 2:**
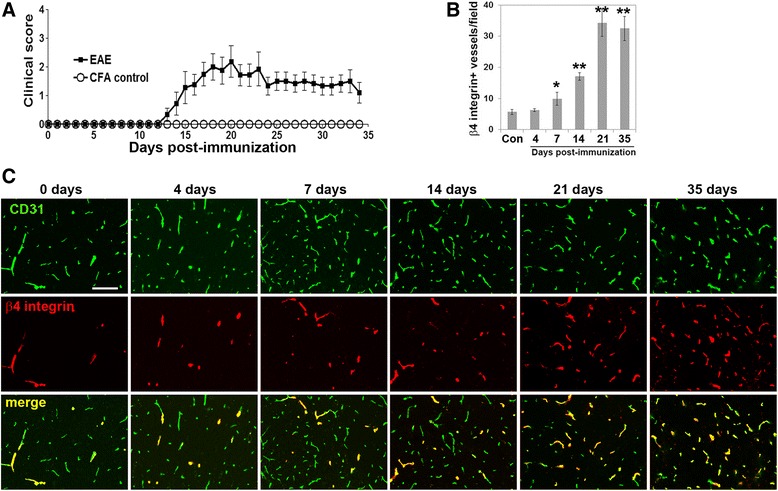
Vascular β4 integrin expression is strongly upregulated during the development of EAE. **a** Time course of increasing EAE severity (clinical score) with time post-immunization. **b** Quantification of the number of blood vessels expressing β4 integrin in frozen sections of the medulla oblongata during EAE progression. Note that significant upregulation of endothelial β4 integrin was detected 7 days post-immunization and peaked after 21 days. *p < 0.05, **p < 0.01. **c** Frozen sections of the medulla oblongata at different time points of EAE progression were dual-stained using antibodies specific for the endothelial marker CD31 (AlexaFluor-488, green) and β4 integrin (Cy3, red). Scale bar = 100 μm. Note that in the disease-free brain (0 days), β4 integrin was expressed by only a fraction of CD31-positive vessels, but the number of vessels expressing β4 integrin gradually increased with EAE progression, such that by the acute stage of EAE (day 21), the vast majority of blood vessels expressed β4 integrin, and this expression was maintained high through to the chronic stage (day 35)

Previous studies have described strong upregulation of β4 integrin on cerebral vessels in two inflammatory models: cerebral aneurysms and the GFAP-IL6 transgenic mouse [23, 24]. To determine whether cerebrovascular levels of β4 integrin are also upregulated in neuroinflammatory demyelinating disease, we studied this process in the experimental autoimmune encephalomyelitis (EAE) mouse model of chronic progressive multiple sclerosis (MS). EAE was induced in 8–10-week-old female C57Bl/6 mice by immunization with MOG_35–55_ peptide, as previously described [38]. In keeping with findings from our lab and others [31, 32, 38], the mice began developing clinical signs 12 days post-immunization and the disease gradually got more severe over time. Clinical severity peaked around 21 days (acute symptomatic phase), and improved slightly thereafter, but never completely recovered at the experimental endpoint of 35 days (chronic symptomatic phase) (Fig. 2a). In this model, robust inflammation is observed in the hindbrain, particularly in the medulla oblongata and cerebellum areas [31, 32, 38]. To examine whether β4 integrin expression levels are regulated during EAE progression, we performed CD31/β4 integrin dual-IF staining on frozen brain sections at 0, 4, 7, 14, 21, and 35 days post-immunization. As shown in Fig. 2b, c, under control conditions (day 0), β4 integrin was expressed by only a small fraction of cerebral blood vessels in the medulla region of the brain, but as EAE developed, an increasing number of blood vessels showed expression of the β4 integrin subunit, such that by the acute stage of EAE (day 21), the vast majority of blood vessels expressed β4 integrin, and this expression was maintained high through to the chronic stage (day 35). Quantification revealed that compared to control (disease-free) conditions, significant upregulation of endothelial β4 integrin was first detected 7 days post-immunization (9.9 ± 2.1 compared to 5.7 ± 0.8 β4 integrin + vessels per field of view (FOV) *p* < 0.05); this reached a peak after 21 days (34.3 ± 4.4 compared to 5.7 ± 0.8 β4 integrin + vessels per FOV, *p* < 0.01) and was still maintained 35 days post-immunization (32.5 ± 3.9 compared to 5.7 ± 0.8 β4 integrin + vessels per FOV, *p* < 0.01 (Fig. 2b).

### Genetic deletion of endothelial β4 integrin results in worse clinical disease and neuroinflammation in EAE

**Fig. 3:**
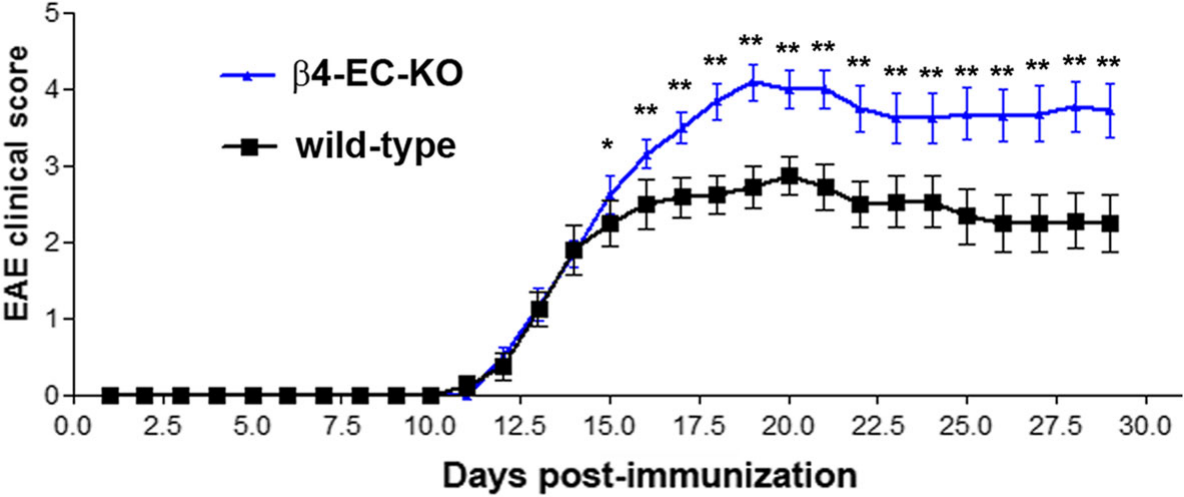
The impact of genetically deleting endothelial β4 integrin on clinical severity in EAE. The progression of EAE in the β4-EC-KO and WT littermate control mice was evaluated by measuring clinical score on daily intervals. All points represent the mean ± SD (*n* = 16 mice per strain). Note that while the β4-EC-KO mice showed the same time of disease onset as the WT littermates, over time, they developed significantly worse clinical disease, resulting in increased mean clinical score for the duration of the experiment. This result was confirmed in three separate experiments. *p < 0.05, **p < 0.01

Previous work has shown that β4 integrin plays an essential protective role in the skin by promoting mechanical and barrier integrity in epidermal cells, such that β4 integrin global KO mice show a perinatal lethal phenotype as a result of epidermal detachment and severe skin blistering [25–27]. To examine whether absence of endothelial β4 integrin has any functional consequences on the vascular integrity of cerebral blood vessels, we used a Cre-Lox approach to generate mice lacking β4 integrin specifically in endothelial cells (β4-EC-KO strain), by crossing floxed β4 integrin mice with Tie2-Cre transgenic mice, as previously described [22]. The β4-EC-KO mice are viable and fertile and show no obvious defects in developmental angiogenesis or vascular function in the adult [22] and thus are amenable to experimental analysis. EAE was induced in the 8–10-week-old female β4-EC-KO and WT littermate mice and disease progression compared (Fig. 3). This showed that while the β4-EC-KO mice showed the same time of onset of EAE as the WT littermates, over time, they developed significantly worse clinical disease, resulting in an increased mean clinical score at the peak of disease, and this elevated mean clinical score was maintained all the way from a relatively early stage of disease (day 15 post-immunization), through the acute stage (day 20–day 22) right up to day 30 (chronic stage) (Fig. 3). This result was confirmed in three separate experiments.

**Fig. 4:**
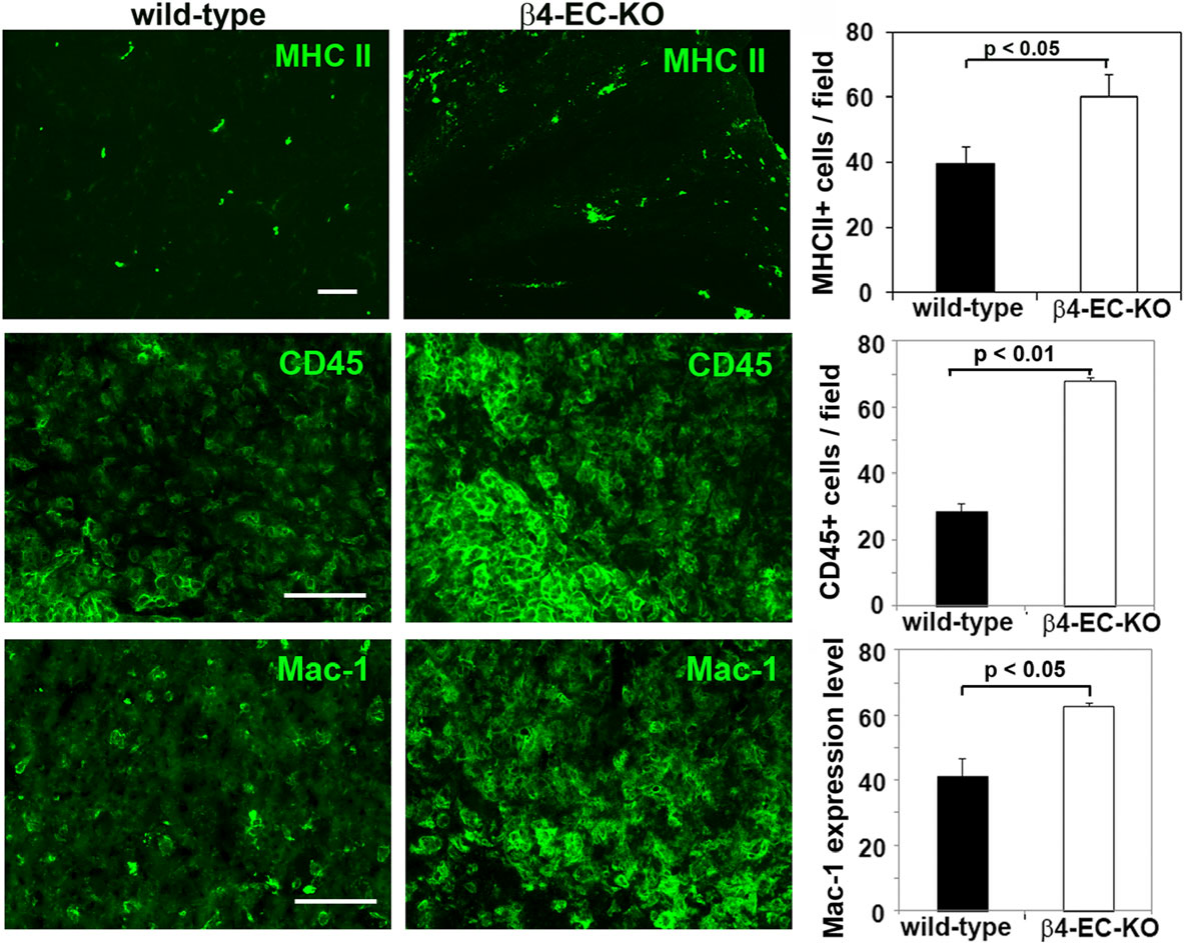
The impact of genetically deleting endothelial β4 integrin on neuroinflammation in EAE. Frozen brain sections taken from the β4-EC-KO and WT littermate control mice at the acute stage of EAE (day 21) were stained using antibodies specific for the inflammatory leukocyte markers MHC II, CD45, and Mac-1. Data points represent the mean ± SEM of events observed in the medulla oblongata (*n* = 4 mice). Scale bar = 100 μm. Note that quantification of all three markers revealed that β4-EC-KO brains contained increased numbers of MHC II+ and CD45+ inflammatory leukocytes, as well as increased expression level of the microglial/macrophage marker Mac-1

To investigate how lack of endothelial β4 integrin affects neuroinflammation in the EAE model, we used IF to examine the distribution of three markers of inflammatory cells in the medulla region of the brain: MHC class II, CD45, and Mac-1. As shown in Fig. 4, quantification of all three markers at the acute phase of EAE (day 21) revealed that compared to the WT controls, β4-EC-KO brains contained increased numbers of MHC II+ (60.3 ± 6.8 vs. 39.5 ± 5.4 MHC II+ cells per FOV, *p* < 0.05) and increased numbers of CD45+ inflammatory leukocytes (67.8 ± 1.2 vs. 28.5 ± 2.4 CD45+ cells per FOV, *p* < 0.01), as well as increased expression level of the microglial/macrophage marker Mac-1 (62.9 ± 1.1 vs. 41.1 ± 5.7 expression level per FOV, *p* < 0.05). Similar observations were also found in the cerebellum, another area showing marked leukocyte infiltration in the EAE model. Thus, in this EAE model, absence of endothelial β4 integrin resulted in worse clinical disease, correlating with increased neuroinflammation.

### In EAE, cerebral blood vessels in β4-EC-KO show enhanced vascular leakage, correlating with reduced expression of tight junction proteins

**Fig. 5:**
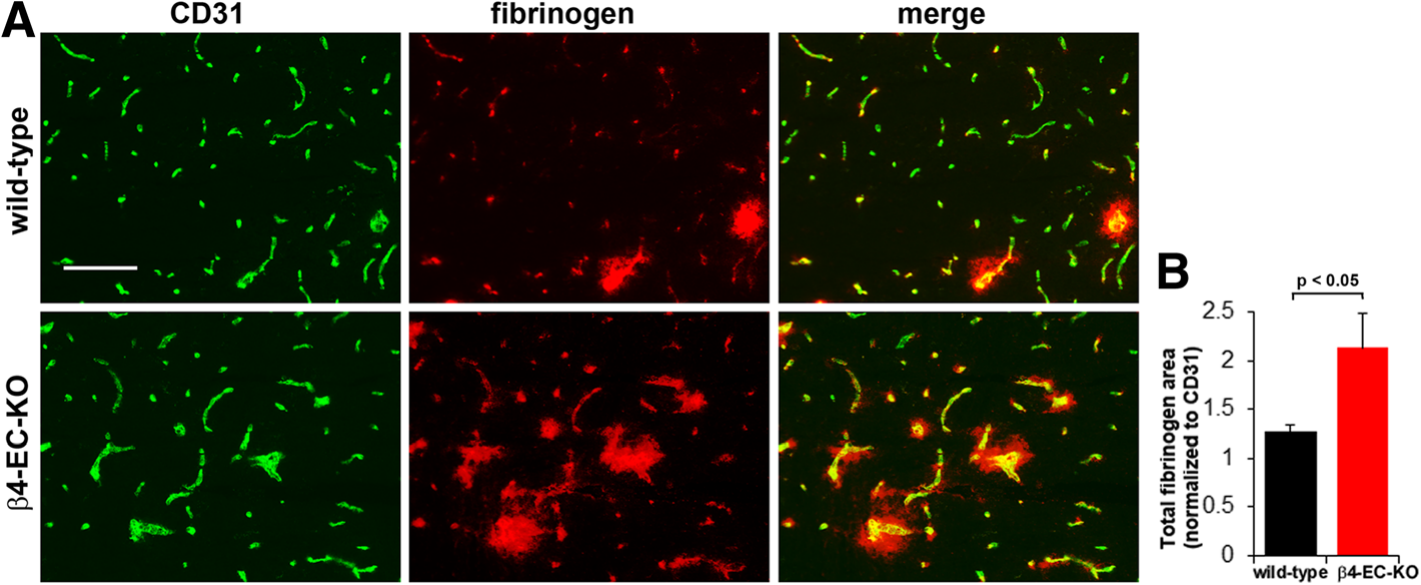
Evaluating the impact of genetically deleting endothelial β4 integrin on BBB integrity in EAE. **a** Frozen sections of the medulla oblongata taken from the β4-EC-KO and WT littermate control mice at the acute stage of EAE (day 21) were dual-stained using antibodies specific for the endothelial marker CD31 (AlexaFluor-488, green) and fibrinogen (Cy3, red). Scale bar = 100 μm. **b** Quantification of fibrinogen leakage in the β4-EC-KO vs. WT littermate mice. Data points represent the mean ± SEM of events observed in the medulla oblongata (*n* = 4 mice). Scale bar = 100 μm. Note that that in most blood vessels, fibrinogen staining was localized to within the lumen of blood vessels. However, in some blood vessels, fibrinogen staining extended beyond the vascular margin, creating a fuzzy leakage pattern, indicative of loss of vascular integrity. Also note that in the β4-EC-KO mice, fibrinogen leakage was noticeably worse than the WT controls, both in the number and extent of leaky vessels, and this was confirmed by quantification

In light of the barrier-stabilizing role of α6β4 in the skin [25–27], and our data showing worse clinical disease and leukocyte infiltration in the EAE model, we next examined whether lack of endothelial β4 integrin results in weakened vascular integrity of cerebral blood vessels. Using fibrinogen leakage as a marker of BBB breakdown, CD31/fibrinogen dual-IF of the medulla showed that in the majority of blood vessels, fibrinogen staining was localized to within the lumen of blood vessels (Fig. 5), consistent with previous findings [38]. However, in some blood vessels during the acute stage of EAE, fibrinogen staining also extended beyond the vascular margin, creating a fuzzy leakage pattern around some blood vessels, indicating vascular breakdown. Interestingly, the pattern of fibrinogen leakage appeared noticeably worse in the β4-EC-KO mice compared to the WT controls, both in terms of the number of leaky vessels and the extent of leakage. Fibrinogen leakage was quantified using ImageJ analysis software and revealed that the extent of fibrinogen leakage was significantly greater in the β4-EC-KO mice compared to the WT littermate controls (2.13 ± 0.35 compared to 1.27 ± 0.06 fibrinogen/CD31 ratio as described in the “Methods” section, *p* < 0.05, Fig. 5b).

**Fig. 6:**
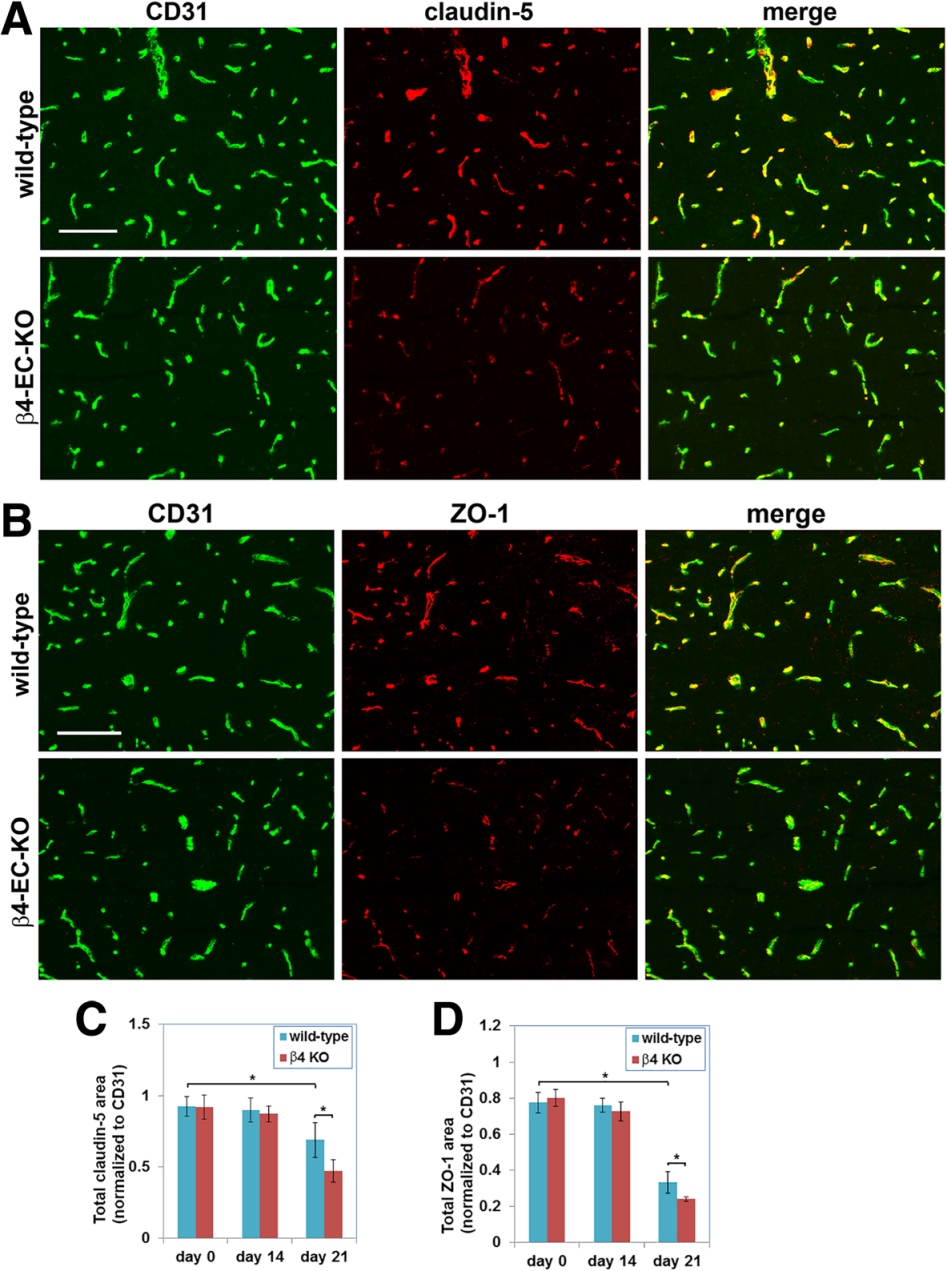
Evaluating the impact of genetic deletion of endothelial β4 integrin on endothelial tight junction protein expression in EAE. **a**, **b** Frozen sections of the medulla oblongata taken from the β4-EC-KO and WT littermate control mice at the acute stage of EAE (day 21) were dual-stained using antibodies specific for the endothelial marker CD31 (AlexaFluor-488, green) and claudin-5 (Cy3, red) or CD31 (AlexaFluor-488, green) and ZO-1 (Cy3, red) in A and B, respectively. Scale bar = 100 μm. **c**, **d** Quantification of endothelial expression of claudin-5 (**c**) and ZO-1 (**d**) in the β4-EC-KO vs. WT littermate mice. Data points represent the mean ± SEM of events observed in the medulla oblongata (*n* = 4 mice). Note that in the WT mice, endothelial levels of claudin-5 and ZO-1 were markedly reduced in the acute phase of EAE (day 21), and at this phase of disease, expression levels in the β4-EC-KO mice were significantly reduced compared to their WT littermates

As cerebral blood vessels rely on expression of tight junction proteins such as claudin-5 and ZO-1 to maintain high vascular integrity [7, 9, 11], we next examined whether expression of these proteins is altered in cerebral blood vessels of the β4-EC-KO mice during EAE by performing dual-IF of CD31/claudin-5 or CD31/ZO-1 on brain sections taken from the β4-EC-KO or WT littermate mice during the acute stage of EAE. This showed that consistent with previous reports, claudin-5 and ZO-1 co-localize very tightly with the endothelial cell marker CD31 on all blood vessels (Fig. 6). Importantly, no obvious difference in the expression level of claudin-5 or ZO-1 was detected between the β4-EC-KO or WT littermate mice under disease-free conditions or in the pre-symptomatic phase (14 days post-immunization) (Fig. 6c, d). However, during the acute phase of EAE (21 days post-immunization), the vascular expression levels of claudin-5 and ZO-1 were significantly reduced compared to the disease-free control levels, and interestingly at the acute phase of disease, levels in the β4-EC-KO mice were markedly lower than those in the WT littermates. By quantifying the ratio of tight junction protein area to CD31 area (to allow for variations in vascular density between different FOVs), this revealed that in the WT mice, vascular claudin-5 levels were reduced from 0.92 ± 0.06 under control conditions to 0.69 ± 0.12 in the acute phase of EAE (*p* < 0.05) and at this stage of EAE, claudin-5 levels in the β4-EC-KO mice were significantly lower than their WT littermates (0.47 ± 0.07 vs. 0.69 ± 0.12, *p* < 0.05). In a similar manner, vascular expression of ZO-1 in the brains of the WT mice was reduced from 0.77 ± 0.05 under control conditions to 0.33 ± 0.06 in the acute phase of EAE (*p* < 0.05), and at this stage of EAE, ZO-1 levels in the β4-EC-KO mice were significantly lower than their WT littermates (0.24 ± 0.01 vs. 0.33 ± 0.06, *p* < 0.05). Thus, during the acute stage of EAE, cerebrovascular levels of both claudin-5 and ZO-1 were significantly lower in the β4-EC-KO mice than their WT littermates.

### Under pro-inflammatory conditions, β4 integrin null BECs show reduced levels of claudin-5 and ZO-1

**Fig. 7:**
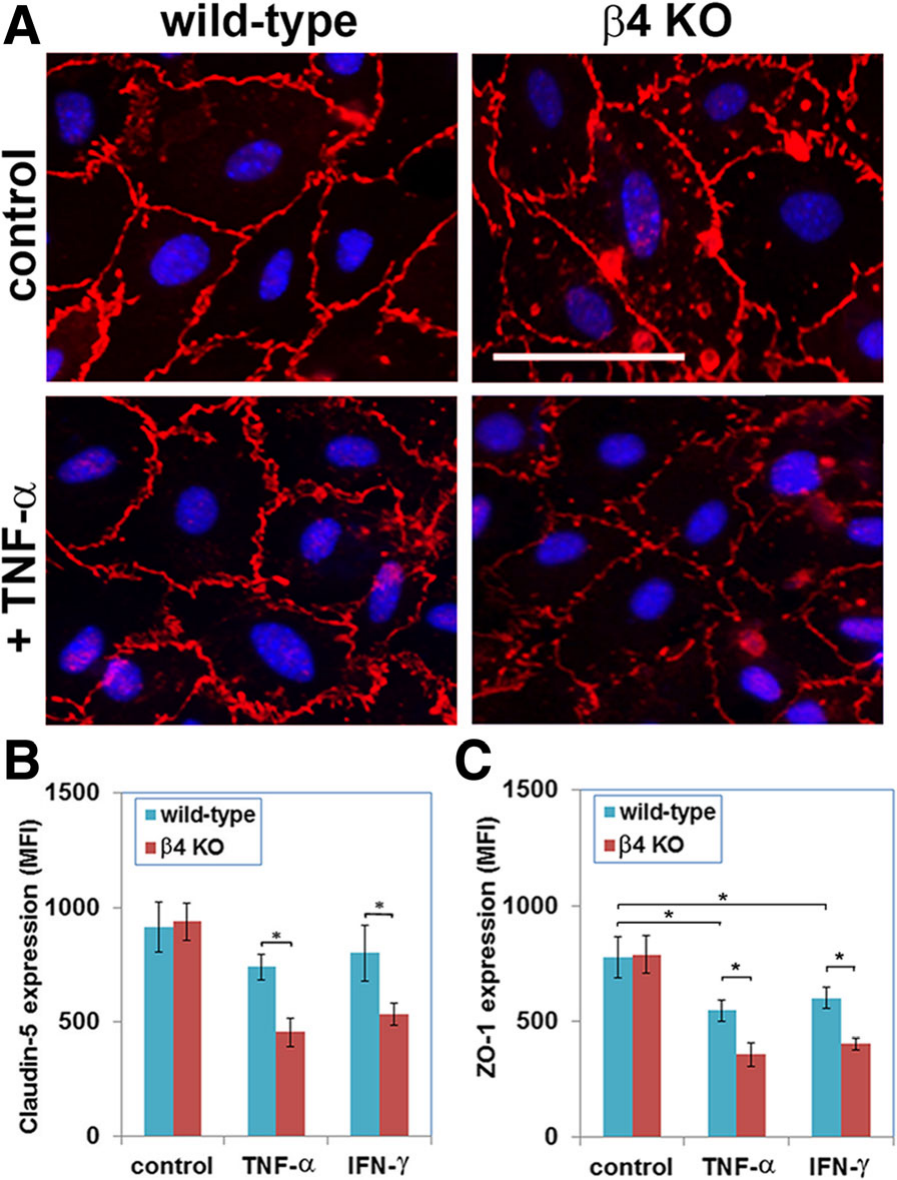
Evaluating endothelial tight junction protein expression in primary brain endothelial cells (BECs). **a** BECs from the β4-EC-KO and WT littermate control mice were cultured on laminin in the presence or absence of TNF-α, and claudin-5 expression was examined by IF. **b**, **c** Quantification of claudin-5 (**b**) or ZO-1 (**c**) expression by β4 integrin null and WT BECs under pro-inflammatory conditions (treatment with TNF-α or IFN-γ). All points represent the mean ± SEM of the mean fluorescent intensity (MFI) of three separate experiments. Note that while ZO-1 expression in WT BECs was significantly reduced by TNF-α and IFN-γ, expression of claudin-5 was not significantly affected by either of these cytokines. Furthermore, compared to WT BECs, while β4 integrin KO cells expressed equivalent levels of claudin-5 and ZO-1 under basal conditions, after exposure to TNF-α or IFN−γ, β4 integrin KO BECs expressed significantly lower levels of claudin-5 and ZO-1. *p < 0.05

Following on from our observation that in EAE-affected brain, blood vessels in the β4-EC-KO mice appear to show greater loss of claudin-5 than the WT controls, we wanted to test directly whether absence of β4 integrin impacts the expression of claudin-5 or ZO-1 at cell-cell junctions. To examine this more closely at the cellular level, we isolated primary brain endothelial cells (BECs) from the β4-EC-KO and WT littermate mice and cultured them on laminin (the ECM ligand for α6β4 integrin) until confluent, at which point TNF-α was added to mimic inflammatory conditions. Forty hours later, claudin-5 expression was examined by immunocytochemistry. As shown in Fig. 7a, under control conditions, β4 integrin KO and WT BECs expressed equivalent levels of claudin-5. However, after culture with TNF-α, β4 integrin KO BECs showed much weaker expression of claudin-5 at cell-cell junctions. To confirm this result, we examined claudin-5 expression by flow cytometry as previously described [14]. This showed that while ZO-1 expression in WT BECs was significantly reduced by TNF-α (from 776 ± 59 under control conditions to 546 ± 46.1, *p* < 0.05) and IFN-γ (from 776 ± 59 under control conditions to 601 ± 47.3, *p* < 0.05), expression of claudin-5 was not significantly affected by either of these cytokines. Interestingly, while β4 integrin KO and WT BECs expressed equivalent levels of claudin-5 and ZO-1 under basal conditions, after exposure to TNF-α or IFN-γ, β4 integrin KO cells expressed significantly lower levels of claudin-5 (with TNF-α treatment, 454 ± 63.1 vs. 739 ± 54.2, *p* < 0.05 and with IFN-γ treatment, 532 ± 49.3 vs. 801 ± 121.4, *p* < 0.05) (Fig. 7b) and ZO-1 (with TNF-α treatment, 357 ± 49.8 vs. 546 ± 46.2, *p* < 0.05 and with IFN-γ treatment, 404 ± 25.5 vs. 601 ± 47.1, *p* < 0.05) (Fig. 7c). These in vitro results support our in vivo observations and suggest that β4 integrin confers vasculo-protection in part by promoting vascular integrity via stabilization of the tight junction proteins claudin-5 and ZO-1.

## Discussion

The goal of this study was to examine the contribution of endothelial α6β4 integrin to BBB integrity during neuroinflammatory disease. Previous studies have described marked upregulation of α6β4 integrin expression on cerebral blood vessels during neuroinflammation [23, 24]. Taken with its essential role in the maintenance of epidermal integrity in skin [25–27], this prompted us to wonder if α6β4 integrin induction on brain endothelium might be part of a physiological adaptive response in an attempt to secure BBB integrity under neuroinflammatory conditions. In this study, we set out to answer this question by using the mouse EAE model of chronic progressive MS. Our main findings were (i) α6β4 integrin is strongly upregulated on all cerebral blood vessels during the progression of EAE; (ii) genetic deletion of endothelial β4 integrin (β4-EC-KO mice) results in worse clinical disease in EAE, correlating with increased leukocyte infiltration into the CNS; (iii) during EAE, cerebral blood vessels in the β4-EC-KO mice show enhanced vascular leakage of the plasma protein fibrinogen; (iv) this vascular leak correlates with reduced expression of the tight junction proteins claudin-5 and ZO-1 in the blood vessels of the β4-EC-KO mice; and (v) parallel in vitro studies showed that under pro-inflammatory conditions, primary cultures of β4KO BECs also showed reduced expression of claudin-5 and ZO-1 expression. Taken together, these studies support the notion that α6β4 integrin upregulation is an inducible protective mechanism that stabilizes the BBB during neuroinflammatory conditions.

### The role of cell adhesion molecules in maintaining BBB integrity

Constituent cells of the BBB adhere to vascular basal lamina proteins such as laminin, collagen IV, fibronectin, and heparin sulfate proteoglycan (HSPG) predominantly via cell surface receptors of the integrin family [13, 37]. These interactions between ECM proteins and their cognate receptors regulate not just cell adhesion but also many aspects of cell behavior including survival, proliferation, migration, differentiation, and stability, via well-defined integrin-mediated intracellular signaling pathways [39, 40]. Endothelial cells form the inner layer of blood vessels and, as such, represent the first layer of resistance to the passage of cells (e.g., inflammatory leukocytes) or substances attempting to pass through the BBB into the cerebral parenchyma. Current evidence now suggests that the molecular basis of the BBB depends on three main factors: (i) inter-endothelial tight junction proteins, (ii) endothelial adhesion to the ECM proteins of the underlying vascular basal lamina, and (iii) the influence of astrocyte end-feet and pericytes [11–14]. Thus far, most attention has focused on the role of tight junction proteins in establishing and maintaining this barrier, while relatively few studies have examined the role of cell adhesion mechanisms in regulating BBB formation and integrity. However, a growing number of studies suggest that ECM-integrin interactions are not just essential for vascular formation and remodeling, but also play an important role in mediating BBB integrity. While it has been known for some time that laminin imparts an important differentiation and stabilizing influence on endothelial cells [15–19], more recent studies show that it is also required for vascular integrity, as genetic deletion of laminin in astrocytes or pericytes results in defective BBB integrity [20, 21]. In support of this important role for laminin in stabilizing the BBB, a number of studies have shown that loss of the astrocyte laminin receptor, dystroglycan, accompanies vascular leakage at the BBB and that prevention of this loss ameliorates BBB breakdown [41–43]. Furthermore, several years ago, we showed that pharmacological inhibition of the β1 class of integrins leads to increased microvascular permeability in the brain as well as an in vitro endothelial permeability system and that increased vascular permeability correlates with reduced endothelial expression of the tight junction protein claudin-5 [14]. The findings of our current study extend these findings by providing more support for a critical role for laminin and its cellular receptors in maintaining BBB properties in cerebral blood vessels. To our knowledge, these studies are the first to demonstrate an active upregulation of an adhesive mechanism (α6β4 integrin) in response to a potential threat of BBB breakdown.

### The unique properties of β4 integrin make it well suited to confer enhanced integrity in cerebral blood vessels

Amongst cell adhesion molecules, the β4 integrin is unique in several ways. First, while most endothelial adhesion molecules (several different β1 integrins and dystroglycan) are expressed universally by all types of blood vessel, in the normal CNS, β4 integrin expression is restricted specifically to arterioles [22], but interestingly during neuroinflammatory conditions, β4 integrin is strongly upregulated by all types of blood vessel [23, 24], demonstrating a unique response property of this integrin. Second, the cytoplasmic domain of the β4 integrin subunit (~ 1000 amino acids) is much longer and quite different from the highly conserved cytoplasmic domain other β integrin subunits (~ 50 amino acids), implying greater potential for unique interactions with cytoskeletal adaptor proteins and intracellular signaling pathways [44]. This idea is supported by the finding that while the cytoplasmic domain of most integrin β subunits (e.g., β1, β3, and β5) interacts with the actin cytoskeleton [37, 40], the β4 integrin cytoplasmic domain interacts specifically with intermediate filament proteins such as vimentin and desmin [45, 46]. Third, in the epidermis, β4 integrin plays an essential role in promoting mechanical stability and barrier integrity, such that mutation or deletion of β4 integrin results in skin fragility and blistering in humans and perinatal lethality in β4 integrin global KO mice [25–27]. Based on this collective evidence, we postulate that endothelial cells of the BBB respond to the threat of leukocyte infiltration by increasing β4 integrin expression, thus strengthening the transmembrane linkage connecting the basal lamina laminin with the cytoskeletal proteins of endothelial cells, resulting in enhanced BBB integrity.

### Does upregulation of endothelial β4 integrin hold therapeutic potential?

Our studies revealed that absence of endothelial β4 integrin had no impact on the initial timing of onset of EAE, consistent with a lack of β4 integrin expression on microvascular endothelium at this early time point, but as EAE developed, β4 integrin was upregulated on vascular endothelium to provide significant protection in the WT mice compared to β4-EC-KOs. These findings suggest that factors that enhance endothelial β4 integrin expression might afford increased protection against neuroinflammation. Importantly, several studies have shown that statins, cholesterol-lowering drugs that exert potent vasculo-protective and anti-inflammatory effects [47–49], are strong inducers of β4 integrin gene expression in endothelial cells. This was first demonstrated in a microarray study of human umbilical vein endothelial cells (HUVECs), in which from a panel of more than 6000 candidate genes, β4 integrin stood out as being the gene which showed the greatest (30-fold) increase following treatment with atorvastatin or pitavastatin [50]. Since this time, other studies have confirmed this finding [51, 52] and also shown that statins prevent breakdown of cerebrovascular basal lamina proteins in ischemic stroke, consistent with a protective role in preserving BBB integrity [53]. As the beneficial anti-inflammatory effects of statins are well proven [47–49], as well as their ability to suppress EAE progression [54–56], it is tempting to speculate that some of this protection may be in part due to enhanced endothelial β4 integrin expression. In subsequent experiments, we will test this hypothesis by treating mice with atorvastatin to stimulate endothelial β4 integrin expression on cerebral blood vessels and compare the anti-inflammatory effects of atorvastatin in β4-EC-KO mice and WT littermates. If our prediction is correct, a significant part of the protection conferred by atorvastatin will be lost in β4-EC-KO mice. In a more direct approach, we also plan to generate a novel transgenic mouse strain in which β4 integrin is specifically over-expressed in endothelial cells and evaluate whether this strain is protected against EAE.

## Conclusions

The goal of this study was to examine the contribution of endothelial α6β4 integrin to BBB integrity in neuroinflammatory disease. IF analysis showed that endothelial β4 integrin expression was markedly upregulated during EAE progression, such that by the acute stage of EAE (day 21), the vast majority of blood vessels expressed β4 integrin. In the EAE model, while the β4-EC-KO mice showed the same time of disease onset as the WT littermates, they developed significantly worse clinical disease over time, resulting in increased clinical score at the peak of disease and maintained elevated thereafter. Consistent with this, the β4-EC-KO mice showed enhanced levels of leukocyte infiltration and BBB breakdown and also displayed increased loss of the endothelial tight junction proteins claudin-5 and ZO-1. Under pro-inflammatory conditions, primary cultures of β4KO BECs also showed increased loss of claudin-5 expression. Taken together, our data suggest that α6β4 integrin upregulation is an inducible protective mechanism that stabilizes the BBB during neuroinflammatory conditions.

## Abbreviations

BBB: Blood-brain barrier
BEC: Brain endothelial cells
CFA: Complete Freund’s adjuvant
CNS: Central nervous system
Dual-IF: Dual immunofluorescence
EAE: Experimental autoimmune encephalomyelitis
ECM: Extracellular matrix
FOV: Field of view
HSPG: Heparan sulfate proteoglycan
IFN-γ: Interferon gamma
KO: Knockout
MHC: Major histocompatibility complex
MOG: Myelin oligodendrocyte protein
MS: Multiple sclerosis
SEM: Standard error of the mean
SMA: Smooth muscle actin
TNF-α: Tumor necrosis factor-alpha
WT: Wild-type
ZO-1: Zonula occludens-1
